# Hetero-Interfaces on Cu Electrode for Enhanced Electrochemical Conversion of CO_2_ to Multi-Carbon Products

**DOI:** 10.1007/s40820-022-00879-5

**Published:** 2022-06-14

**Authors:** Xiaotong Li, Jianghao Wang, Xiangzhou Lv, Yue Yang, Yifei Xu, Qian Liu, Hao Bin Wu

**Affiliations:** Institute for Composites Science Innovation (InCSI) and State Key Laboratory of Silicon Materials, School of Materials Science and Engineering, Zhejiang University, Hangzhou, 310027 People’s Republic of China

**Keywords:** CO_2_ reduction reaction, Metal–organic frameworks, Copper, Hetero-interfaces, Multi-carbon products

## Abstract

Electrochemical CO_2_ reduction reaction (CO_2_RR) to multi-carbon products would simultaneously reduce CO_2_ emission and produce high-value chemicals. Herein, we report Cu electrodes modified by metal–organic framework (MOF) exhibiting enhanced electrocatalytic performance to convert CO_2_ into ethylene and ethanol. The Zr-based MOF, UiO-66 would in situ transform into amorphous ZrO_x_ nanoparticles (a-ZrO_x_), constructing a-ZrO_x_/Cu hetero-interface as a dual-site catalyst. The Faradaic efficiency of multi-carbon (C2+) products for optimal UiO-66-coated Cu (0.5-UiO/Cu) electrode reaches a high value of 74% at − 1.05 V versus RHE. The intrinsic activity for C2+ products on 0.5-UiO/Cu electrode is about two times higher than that of Cu foil. In situ surface-enhanced Raman spectra demonstrate that UiO-66-derived a-ZrO_x_ coating can promote the stabilization of atop-bound CO* intermediates on Cu surface during CO_2_ electrolysis, leading to increased CO* coverage and facilitating the C–C coupling process. The present study gives new insights into tailoring the adsorption configurations of CO_2_RR intermediate by designing dual-site electrocatalysts with hetero-interfaces.

**Supplementary Information:**

The online version contains supplementary material available at 10.1007/s40820-022-00879-5.

## Highlights

- Dual-site electrocatalysts with in situ formed metal-oxide interfaces are proposed and demonstrated to enhance the formation of multi-carbon products from electrochemical CO_2_ reduction.
- The 0.5-UiO/Cu delivers a high C2+ Faradaic efficiency of over 74% with notably boosted formation rate and durability, surpassing most reported electrocatalysts.
- A unique mechanism of UiO-66 to induce in situ reconstruction of Cu surface and formation of UiO-66-derived amorphous ZrO_x_/Cu interface is uncovered, which improves the selectivity and productivity toward C2+ products.

**Supplementary Information:**

The online version contains supplementary material available at 10.1007/s40820-022-00879-5.

## Graphical abstract

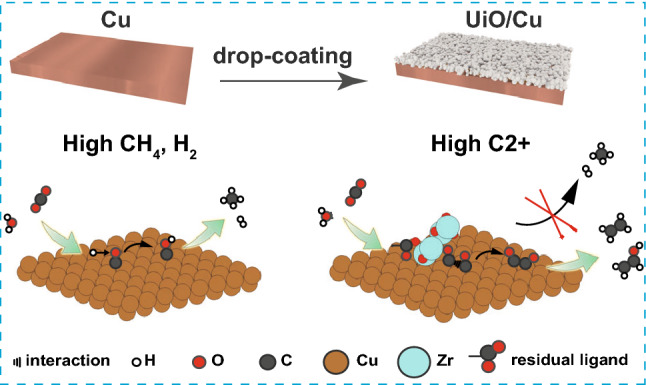

## Introduction

The exploitation and utilization of fossil fuels have led to excessive emissions of CO_2_, which break the carbon cycle in nature and cause serious environmental problems [1]. Hence, it is urgent to develop efficient technologies to convert CO_2_ into valuable fuels and chemicals, especially into C2+ products with high energy density and high value [2–7]. Among all potential technologies, CO_2_ reduction reaction (CO_2_RR) driven by renewable electric energy is practically appealing to realize a benign closed-carbon cycle. However, tremendous challenges, such as high overpotentials, low selectivity, and unsatisfactory long-term stability, have limited the large-scale application of CO_2_RR. Designing advanced electrocatalysts with high performance is the key to overcome these obstacles.

Cu-based materials, with a proper CO adsorption energy, are the most popular catalysts that can convert CO_2_ into C2+ products with non-negligible selectivity [3, 8, 9]. However, unitary Cu-based catalysts generally show unsatisfied selectivity toward C2+ products due to the limitation of scaling relations [10–12]. Developing dual-site catalysts by incorporating the second component, either organic or inorganic moieties, offers new freedom to regulate the adsorption energies of intermediates and modulate the product distribution. For instance, Cu catalysts modified by organic species such as amino acid [13] and polyamine [14–16] exhibited excellent catalytic performance for C2+ products. Cu-based bimetallic catalysts with Bi, Au, Ru, and Pd as second binding sites can tune the adsorption of CO* and promote the conversion of CO_2_ to alcohols [17–19]. Introducing metal elements such as Al with empty orbital to the Cu oxide can stabilize the positive valence state of Cu which is beneficial for the production of C_2_H_4_ [20]. Moreover, metal oxides, such as zinc oxide [21–24], ceria [25–28], alumina [29], zirconia [30], and silica [31] have also been utilized to construct hetero-interfaces with Cu to promote the C2+ selectivity of Cu by multiple interfacial effects [32, 33]. Therefore, we envisage that using proper promoters to active CO_2_ molecules or to stabilize the adsorbed CO* intermediates on Cu electrodes would increase the coverage of CO* and facilitate the subsequent dimerization into C2+ products [16, 34].

Metal–organic frameworks (MOFs) with abundant coordinatively unsaturated sites (CUSs) and porous structure are promising candidates for CO_2_RR [35–37]. The coordinatively unsaturated metal sites in MOFs, with strong Lewis acidity, can interact with O atom in CO_2_ molecules by the Lewis acid–base interaction to activate linear CO_2_ molecules, and thus promote the CO_2_ electrolysis [29, 38, 39]. Inspired by the development of dual-site catalysts to steer the catalytic performance of Cu, we envision that constructing a hetero-interface of MOFs and Cu may enhance the CO_2_RR performance. To check this suppose, UiO-66 with abundant Lewis-acidic Zr sites [40] was used to modify Cu foil by a drop-coating method. During the CO_2_ electrolysis, the UiO-66 nanoparticles transform into a-ZrO_x_ by losing most of the organic ligands, thereby exposing more coordinatively unsaturated Zr sites. In effect, a-ZrO_x_/Cu hetero-interfaces were formed on the electrode during CO_2_ electroreduction. As a result, a significantly enhanced CO_2_RR performance (Faradaic efficiency (FE) of 74% for C2+) has been observed on UiO-66 modified Cu electrode. In situ surface-enhanced Raman spectra revealed that a-ZrO_x_ coating can obviously stabilize the CO* intermediate adsorbed at top sites on the Cu surface, which can increase the coverage of CO* and promote the C–C coupling process.

## Experimental Section

### Chemicals

Zirconium (IV) chloride (ZrCl_4_), benzoic acid (HBC), and dimethyl sulfoxide (DMSO) were purchased from Aladdin. 1,4-dicarboxybenzene (H_2_BDC), potassium bromide (KBr), and deuterium oxide (D_2_O) were purchased from Shanghai Macklin Biochemical. N, N-Dimethylformamide (DMF), methanol (CH_3_OH), ethanol (C_2_H_5_OH), and potassium bicarbonate (KHCO_3_) were purchased from Sinopharm Chemical Reagent. Nafion solution (5% in a mixture of lower aliphatic alcohol and water) was purchased from DuPont. Au@SiO_2_ was purchased from Shiyanjia Lab. Copper foil (Cu, 0.1 mm thickness) was purchased from Tianjin Shentai. All reagents were used directly without further purification.

### Synthesis of Catalysts and Electrode Fabrication

#### Synthesis of UiO-66 Nanoparticles

The UiO-66 nanoparticles were synthesized according to a solvothermal method reported in the literature [41]. In a typical synthesis, ZrCl_4_ (2.06 mmol) and 10 equivalent of HBC with respect to ZrCl_4_ were dissolved in DMF (60 mL) via sonication for 10 min. Then, H_2_BDC (2.06 mmol) with an equimolar ratio with respect to ZrCl_4_ was added to the above solution and dissolved by stirring for 30 min. The obtained solution was transferred into a Teflon liner (100 mL) which was sealed in an autoclave. The tightly capped autoclave was placed in an oven at 120 °C for 24 h. After cooling to room temperature, the precipitate was collected by centrifugation and washed three times with DMF and methanol, respectively. And the as-synthesized UiO-66 was soaked in methanol for three days to exchange DMF. Finally, the as-synthesized UiO-66 was dried at 60 °C overnight.

The size of UiO-66 can be regulated by tuning the equivalent of HBC. Feeding 10, 20 and 30 equivalent of HBC can synthesize UiO-66 with a size of 100, 300 and 600 nm, respectively. Figure S1 revealed the morphology of UiO-66 with different sizes, among which UiO-66 with the size of 100 nm was used as the main sample in our work.

#### Fabrication of UiO-66-modified Cu Electrode

A simple drop-coating method was used to construct the UiO-66-modified Cu electrode. Firstly, the Cu foil was mechanically polished by 3000 mesh silicon carbide paper and ultrasonically washed with DI water for 10 min. Then, the as-synthesized UiO-66 (10 mg, 100 nm) was dispersed in Nafion-alcohol aqueous solution (1 mL). Then, a certain volume of UiO-66 ink was drop-coated on each side of Cu foil and dried in air on a hot plate (110 °C). Through changing the volume of UiO-66 ink, we obtained a series of UiO-66-modified Cu electrodes with different loadings of UiO-66, denoted as X-UiO/Cu (with X mg cm^−2^ of UiO-66). We also obtained UiO-66 with different size modified Cu foil using the same drop-coating method.

The UiO-66 coating on X-UiO/Cu electrode can be removed by sonication in an alcohol-aqueous solution. The reconstructed Cu electrode obtained after sonication was denoted as X-UiO/Cu-bare. Meantime, the powder collected from 0.5-UiO/Cu by sonication was named 0.5-UiO/Cu NPs, which was vacuum dried at 40 °C overnight. We adopted the same strategy as 0.5-UiO/Cu NPs to prepare 0.5-UiO/Cu-CV NPs and 0.5-UiO/Cu-CA NPs which were used to determine the evolution of UiO-66 structure after cyclic voltammetry (CV) process and after chronoamperometry (CA) process.

### Materials Characterizations

X-ray diffraction (XRD) was performed on an X'Pert PRO, PANalytical diffractometer with a Cu Kα radiation source at a scan speed of 2° min^−1^. Scanning electron microscopy (SEM, HITACHI SU8010) equipped with an energy dispersive spectrometer (EDS) was used to confirm the morphologies and element mappings of various electrodes. Transmission electron microscopy (TEM) and high-resolution transmission electron microscope (HR-TEM) images were performed on JEM-2100F. Sample (0.5-UiO/Cu after CO_2_RR) for TEM and HR-TEM measurements were prepared by scraping from the surface of the plane electrode with a blade. X-ray photoelectron spectroscopy (XPS, Thermo Scientific K-Alpha) along with Auger electron spectroscopy was carried out to analyze the surface compositions and chemical states of various electrodes. Fourier transform infrared spectroscopy (FT-IR) was performed on a BRUKER TENSOR 27 instrument.

In situ surface-enhanced Raman measurements were performed in a modified flow cell equipped with a three-electrode system. Electrolysis of CO_2_ was carried out on an electrochemical workstation (CHI 760E) in a CO_2_-saturated 0.1 M KHCO_3_ electrolyte. The Raman signals were recorded on an inVia Reflex Raman microscope (Renishaw) equipped with diode lasers (532 and 633 nm) and a water immersion objective (50×). Each spectrum was obtained using 10% laser power, 10 s of exposure time, and by averaging 3 scans in extended mode. In addition, Au@SiO_2_ nanoparticles were used to amplify the Raman signal. Specifically, 500 μL of Au@SiO_2_ suspension was mixed with 2 μL of Nafion solution. 15 μL of Au@SiO_2_ inks was drop-coated onto the plane electrode. Then, we selected Au@SiO_2_ modified Cu foil (denoted as Cu), Au@SiO_2_ modified 4-UiO/Cu-bare (denoted as UiO/Cu-bare), and Au@SiO_2_ modified 4-UiO/Cu-0.25 (denoted as UiO/Cu) as working electrodes. 4-UiO/Cu-0.25 was prepared by re-coating 0.25 mg cm^−1^ of UiO-66 on the surface of 4-UiO/Cu-bare. The Raman spectra were recorded at open-circuit potential (OCP) and selected potential range from − 0.2 to − 1.2 V versus RHE with a potential interval of 0.2 V. Before CA test, a pre-electrolysis (CV) process was performed to pre-activate working electrodes. Every spectrum was recorded after 5 min of continuous CO_2_ electrolysis.

### Electrochemical Measurements

#### Evaluation of Catalytic Performance

The electroreduction CO_2_ performance of UiO-66-modified Cu electrodes were evaluated in an H-type cell separated by a Nafion proton exchange membrane and equipped with a three-electrode system (Fig. S2). Pt foil was used as the counter electrode, and Ag/AgCl electrode was used as the reference electrode. Two compartments of H-type cell were filled with 30 mL of 0.1 M KHCO_3_ as electrolyte, where the catholyte was saturated with CO_2_ before electrolysis. During the electroreduction CO_2_ process, the flow rate of CO_2_ was kept at a constant rate of 20 mL min^−1^ and the catholyte was continuously stirred at 750 rpm. Chronoamperometry measurements were performed to electrolysis CO_2_ at a selected applied potential ranging from − 0.85 to − 1.1 V versus RHE for 1 h on an electrochemical workstation (Bio-Logic). CV test with the potential range from − 0.41 to − 1.21 V versus Ag/AgCl was used to pre-activate the working electrode before CA test. The gaseous products of electroreduction CO_2_ were quantified by online gas chromatography (GC, FuLi 9790II) equipped with the flame ionization detector (FID) and the thermal conductivity detector (TCD). ^1^H nuclear magnetic resonance with a 600 MHz spectrometer (^1^H NMR, Agilent DD2-600) with a water suppression mode was carried out to quantify the liquid products using DMSO as an internal standard. All potentials in our work were automatically corrected with 85% iR-compensation and converted to reversible hydrogen electrode (RHE) scale according to Eq. (1):

1 $${\text{E }}\left( {vs {\text{RHE}}} \right){ } = {\text{E }}\left( {vs {\text{Ag}}/{\text{AgCl}}} \right) + 0.197{ } + 0.059 \times {\text{pH}}$$

#### Evaluation of the Electrochemically Active Surface Area

Electrochemical double-layer capacitance measurement was carried out to evaluate the surface roughness factors (*R*_f_) and electrochemically active surface area (ECSA) of working electrodes. Cyclic voltammetry measurements (CV) were recorded in the potential range from − 0.14 to − 0.04 V versus OCP in 0.1 M KHCO_3_ for five cycles at scan rates of 20, 40, 60, 80, 100, 150 and 200 mV s^−1^. The differences between anodic and cathodic current densities (Δ*j*) at − 0.09 V versus OCP were calculated and plotted against scan rates. After linear fitting, the slop of Δ*j* versus scan rates was considered as double-layer capacitance (*C*_dl_). *R*_f_ of each catalyst was obtained by calculating the C_dl_ ratio of the modified Cu foil to Cu foil. ECSA of each catalyst was estimated by Eq. (2), where S stands for the geometric area.

2 $$ECSA = R_{f} \times S$$

## Results and Discussion

### Synthesis and Characterizations of MOF-Modified Cu Electrode

**Fig. 1:**
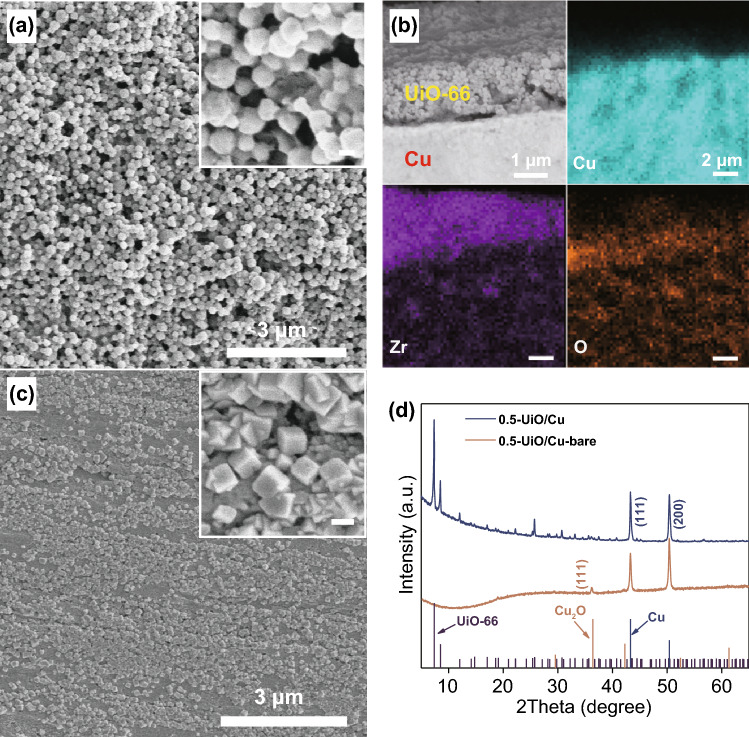
**a** SEM images of 0.5-UiO/Cu (inset: high magnification SEM, the scale bar is 100 nm). **b** Cross-section SEM and EDS elemental mappings of 0.5-UiO/Cu. **c** SEM images of 0.5-UiO/Cu-bare (inset: high magnification SEM, the scale bar is 100 nm) and **d** XRD patterns of 0.5-UiO/Cu and 0.5-UiO/Cu-bare. All samples in Fig. 1 are characterized before CO_2_RR

A simple drop-casting strategy is adopted to construct UiO-66/Cu hetero-interface on the electrode (denoted as X-UiO/Cu, where X is the loading of UiO-66), as shown in Fig. S3. At first, the UiO-66 nanoparticles with a size of 100–200 nm were synthesized by a hydrothermal method (Fig. S4a) and dispersed in a mixture solution of Nafion, alcohol, and deionized (DI) water. The Cu foil was mechanically polished to remove surface impurities (Fig. S4b). The dispersion of UiO-66 was then dropped on the polished Cu foil to form a uniform UiO-66 coating layer on the surface of Cu foil (Fig. 1a). From cross-section scanning electron microscopy (SEM) image and EDS mapping, the porous UiO-66 layer with a thickness of 1.5 μm on top of Cu substrate can be clearly identified (Fig. 1b). The thickness of UiO-66 layer can be adjusted by changing the loading of UiO-66, which might affect the transport of reactants as discussed shortly. Interestingly, the acidic UiO-66 suspension would induce roughening of the Cu surface and formation of well-defined cubic nanoparticles during the preparation of electrode (Fig. 1c). XRD was performed to determine the crystal phase of the cubic nanoparticles. As shown in Fig. 1d, diffraction peaks in the XRD pattern of 0.5-UiO/Cu electrode can be well assigned to UiO-66 and Cu. When UiO-66 layer was removed to expose the reconstructed Cu surface (sample denoted as 0.5-UiO/Cu-bare), an additional diffraction peak corresponding to Cu_2_O emerged, implying that UiO-66 promotes the formation of Cu_2_O on Cu foil by an oxygen-consuming corrosion process. Thus, the well-defined cubic nanoparticles were identified as Cu_2_O.

The Cu LMM Auger electron spectroscopy (AES) of 0.5-UiO/Cu-bare was also carried out to determine the chemical state of Cu. In Fig. S5, the Cu^+^ species was dominant, which was consistent with the results of XRD. Moreover, XPS spectra of C 1*s* and O 1*s* for 0.5-UiO/Cu were presented in Fig. S6a–b to confirm the composition of surface coating. Five peaks with binding energies of 284.8 eV (C–C), 286 eV (C–O), 289.5 eV (C=O), 291.9 eV (CF_2_), and 293.7 eV (CF_3_) were presented in C 1*s* XPS spectrum of 0.5-UiO/Cu [42]. The C–F bonds originated from Nafion, which acted as a binder for UiO-66 nanoparticles. Other bonds were attributed to the ligands of UiO-66. Apart from primary peaks at 535.4 and 533.4 eV, which are attributed to SO_3_H and O–C=O, respectively, secondary peaks assigned to Zr–O and Zr–OH bonds also existed in the O 1*s* XPS spectra [43–45]. In addition, in the Zr 3*d* XPS spectrum of 0.5-UiO/Cu electrode, the binding energies at 186 and 183.6 eV assigned to Zr 3*d*_3/2_ and Zr 3*d*_5/2_ were 0.7 eV higher than that of pristine UiO-66 nanoparticles [46], suggesting charge transfer between Cu and Zr (Fig. S6c–d) [47].

### Performance of Electrochemical CO_2_ Reduction

**Fig. 2:**
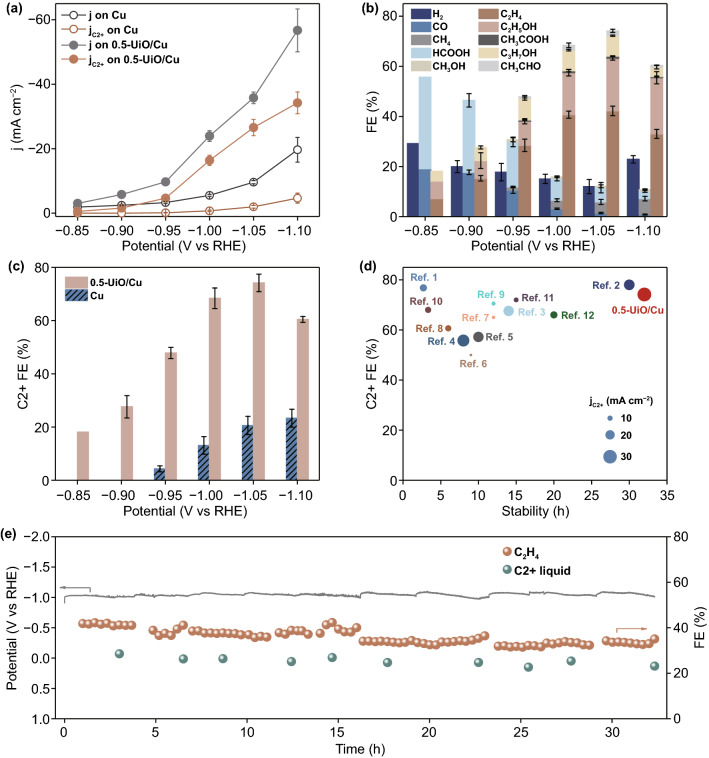
**a** The geometric current densities and partial current densities for C_2+_ products as a function of potential on Cu and 0.5-UiO/Cu. **b** FEs of CO_2_RR and HER products as a function of potential on 0.5-UiO/Cu. **c** FEs of C2+ products on Cu and 0.5-UiO/Cu as a function of potential. **d** Comparison of CO_2_RR performance (including FE and partial current densities for C2+ products and stability) on 0.5-UiO/Cu with state-of-the-art Cu-based catalysts (details shown in Table S2). **e** The chronopotentiometric potential-time curve (left axis) and FEs of C2+ products (right axis) for 0.5-UiO/Cu electrode at − 30 mA cm^−2^ during a stability test of 32 h

CO_2_RR experiments were conducted in an H-type cell with a three-electrode system to explore the catalytic performance of UiO-66-modified Cu electrode. The activities of 0.5-UiO/Cu and Cu foil were assessed by potentiostatic measurements for 1 h at selected potential from − 0.85 to − 1.10 V (versus RHE). All potential in our work were automatically iR-corrected using the electrochemical workstation. As shown in Fig. 2a, 0.5-UiO/Cu gives higher total current densities than that of Cu foil at all tested potentials. Moreover, the partial current densities for C2+ products are obviously increased after modifying the Cu electrode with UiO-66, indicating that UiO-66 may promote the C–C coupling process. Given that the surface reconstruction process induced by UiO-66 coating would hugely increase the surface roughness of Cu foil, we estimated the ECSA corrected current densities of Cu and 0.5-UiO/Cu electrodes. The ECSA of 0.5-UiO/Cu was notably increased to 6.95 times that of pristine Cu foil, as shown in Fig. S7a–b and Table S1, which is consistent with the observed rough surface. Even after deducting the benefit of enlarged surface area, the 0.5-UiO/Cu exhibited a notably increased ECSA-corrected current density of 3.82 mA cm^−2^ for C2+ products at − 1.05 V versus RHE, which is about two times higher than that of Cu foil. The ECSA-corrected current densities for H_2_ and C1 products on 0.5-UiO/Cu were significantly decreased at selected potential range from − 0.85 to − 1.1 V versus RHE. Therefore, the 0.5-UiO/Cu possesses a better intrinsic activity for C2+ products, and the competing reactions toward H_2_ and C1 products are substantially inhibited upon UiO-66 modification (Fig. S7c-d).

Except for the catalytic activity, the UiO-66 modification also has the capability of steering the product distribution of CO_2_RR on Cu (Figs. 2b and S8). The major products of CO_2_RR on Cu foil were altered from H_2_ and C1 products to C2+ products after the modification of UiO-66. The 0.5-UiO/Cu electrode reaches the maximum *FE*_C2+_ of 74% at − 1.05 V versus RHE, which is about 3.6 times higher than that on Cu foil (Fig. 2c). Moreover, the onset potential for C2+ products shifts from − 0.95 V versus RHE on Cu to − 0.85 V versus RHE on 0.5-UiO/Cu, demonstrating the promotion of C–C coupling by UiO-66 modification. Benefiting from the enhanced catalytic activity and selectivity of C2+ products, the formation rate (*r*) for C2+ products on 0.5-UiO/Cu was 13 times higher than that on Cu at − 1.05 V versus RHE, indicating that UiO-66 modification significantly promotes the C2+ production on Cu foil (Fig. S9).

Compared with the state-of-the-art Cu-based catalysts evaluated in H-type cell [10, 15, 27, 48–56], the MOF-modified Cu electrode showed a competitive catalytic performance in terms of activity (− 26.57 mA cm^−2^) and selectivity (74%) toward C2+ products at − 1.05 V versus RHE (Fig. 2d). As shown in Table S2, the formation rate of CO_2_-to-C2+ products on 0.5-UiO/Cu reached 228.08 mol s^−1^ m^−2^, which was higher than most Cu-based catalysts. Moreover, the stability of MOF-modified Cu electrode is also superior to most reported catalysts. As shown in Fig. 2e, the working potential and the FEs for C2+ products were maintained at about − 1.00 V versus RHE and 60%, respectively, during an operating period of 32 h at − 30 mA cm^−2^. The remarkable stability of 0.5-UiO/Cu at a high current density could be related to the high intrinsic stability of the in situ formed a-ZrO_x_/Cu interfacial active sites, in contrast to catalytic sites generated by ex situ approaches such as thermal [57] or plasma [58, 59] pretreatments. All in all, the dramatic enhancement of electrocatalytic CO_2_ conversion toward C2+ products demonstrates the superiority of constructing dual-site catalysts using MOFs for CO_2_RR.

### Real Catalyst Structure of MOF-Modified Cu Electrode

**Fig. 3:**
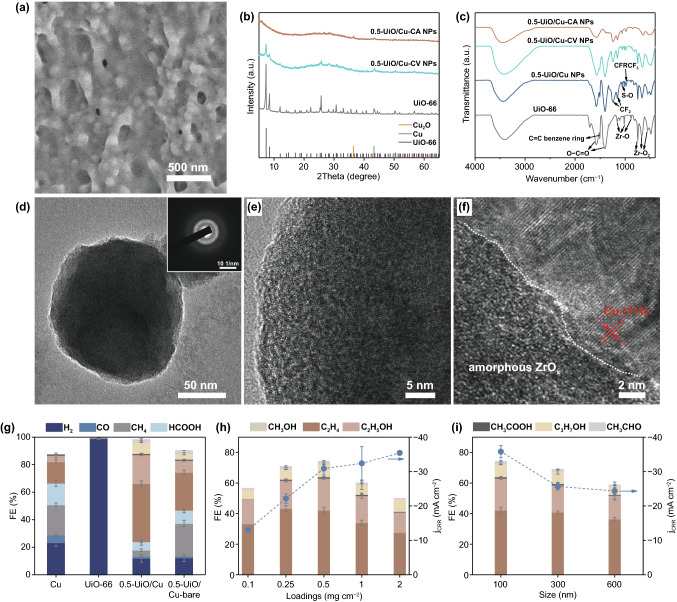
**a** Top-view SEM image of 0.5-UiO/Cu after CO_2_RR at − 1.05 V versus RHE for 1 h. **b** XRD patterns and **c** FT-IR spectra of UiO-66, 0.5-UiO/Cu NPs, 0.5-UiO/Cu-CV NPs, and 0.5-UiO/Cu-CA NPs. **d** TEM image of 0.5-UiO/Cu-CA NPs (inset: the corresponding SAED pattern). **e** HRTEM image of 0.5-UiO/Cu-CA NPs. **f** HR-TEM image of interface structure of 0.5-UiO/Cu after CO_2_RR at − 1.05 V versus RHE for 1 h. The product distribution of HER and CO_2_RR **g** on Cu, UiO-66, 0.5-UiO/Cu and 0.5-UiO/Cu-bare electrodes, **h** on X-UiO/Cu electrodes, and **i** on different size of UiO-66 modified Cu electrodes

Detailed characterizations were conducted to explore the real structure of MOF-modified Cu electrode during CO_2_ electrolysis. SEM images show that the UiO-66 nanoparticles start to collapse and adhere together after CV test and then progress to severe adhesions after CA test (Figs. 3a and S10a). Meanwhile, the reconstructed Cu surface became rougher after CA test, which may lead to more hetero-interfaces and stronger interface interaction (Fig. S10b–c). The morphological evolution of surface coating implies the collapse of UiO-66 structure. Thus, XRD analysis was performed on the surface layer collected from the 0.5-UiO/Cu electrodes after the CV test (denoted as 0.5-UiO/Cu-CV NPs) or the CA test (denoted as 0.5-UiO/Cu-CA NPs). As shown in Fig. 3b, the XRD peaks of UiO-66 diminished after the CV and disappeared after the CA test, indicating that the highly periodic arrangements of UiO-66 were destroyed. The decomposition of UiO-66 was also confirmed by FT-IR analysis of the 0.5-UiO/Cu-CA NPs (Fig. 3c). The peaks at 1578, 1398 and 1504 cm^−1^, which can be assigned to asymmetric stretching, symmetric stretching of carboxylate groups (O–C=O), and vibration of C=C bond in benzene ring, respectively, were almost disappeared after the CA test, indicating the loss of ligands during CO_2_ electrolysis [45, 60]. The peaks located at 980–1220 cm^−1^ in the FT-IR spectra of 0.5-UiO/Cu NPs (surface layer collected from fresh electrode) are attributed to the main vibrational peaks of Nafion binder [61]. Moreover, ex situ XPS was performed on 0.5-UiO/Cu electrode to investigate the change of surface composition during CO_2_RR. As shown in Fig. S11, the signals of C and O assigned to ligands (C=O, C–O, C–C) were obviously weakened while the Zr-O-Zr component was strengthened compared with pristine electrode (Fig. S6), indicating the increased inorganic components and decreased organic components in the surface layer after CO_2_ electrolysis.

TEM was used to investigate the surface layer of the MOF-modified Cu electrode. Figure 3d depicts a typical UiO-66-derived nanoparticle collected from the 0.5-UiO/Cu electrode after CA test, and the corresponding selected area electron diffraction (SAED) pattern (inset of Fig. 3d) and high-resolution TEM (HRTEM) image (Fig. 3e) reveal its amorphous nature. On the basis of above characterizations, we can conclude that the UiO-66 nanoparticles transform into amorphous ZrO_x_ (a-ZrO_x_) nanoparticles with residual ligands during CO_2_RR, forming an a-ZrO_x_-modified Cu electrode. As shown in Fig. 3f, a clear interfacial boundary can be found between a crystalline and an amorphous phase. The lattice fringes with spacing of 0.21 nm in the crystalline region correspond to the (111) plane of Cu, while the amorphous region could be assigned to the ZrO_x_ component. The intimate contact of the two phases forms robust a-ZrO_x_/Cu hetero-interfaces in the 0.5-UiO/Cu electrode during CO_2_RR, which serve as potential catalytic active sites for CO_2_ conversion.

To elucidate the roles of UiO-66 on the CO_2_RR performance, a series of control experiments were performed. Firstly, UiO-66 itself does not exhibit any catalytic performance toward CO_2_RR (Fig. 3g). Secondly, considering that oxide-derived Cu possesses a better catalytic performance for CO_2_RR toward C_2+_ products than conventional Cu [57, 62–64], we assessed the catalytic performance of 0.5-UiO/Cu-bare electrode with Cu_2_O on the surface. As expected, 0.5-UiO/Cu-bare achieved an improved FE for C2+ products at − 1.05 V versus RHE compared with Cu foil (Fig. 3g). However, the FEs of C2+ products on 0.5-UiO/Cu-bare (43%) were still much lower than that on 0.5-UiO/Cu (74%), which confirmed that the in situ formed a-ZrO_x_/Cu interfaces played an important role.

Furthermore, the density of a-ZrO_x_/Cu interface has been adjusted by changing the loading and size of UiO-66. As shown in Fig. 3h, the FEs of C_2+_ products as a function of UiO-66 loadings exhibited a volcano-shaped tendency. Low loading of UiO-66 (such as 0.1-UiO/Cu) may lead to insufficient a-ZrO_x_/Cu interfaces, while thick coating layer may block the mass transport of CO_2_. Thus, 0.5-UiO/Cu with an intermediate loading achieved the optimal selectivity for CO_2_RR toward C2+ products. In addition, the catalytic activities in terms of current density for CO_2_RR on X-UiO/Cu increased with increasing loading of UiO-66, which followed the tendency of ECSA for X-UiO/Cu. In addition, increasing the size of UiO-66 decreased the FE for C2+ products on UiO/Cu electrodes as shown in Fig. 3i, which could be explained by the fewer contact points between the large UiO-66-derived a-ZrO_*x*_ and Cu. Combining characterizations and control experiments, the evolution process of UiO-66 coating under CO_2_ electrolysis is schematically presented in Fig. S12.

### In Situ Spectroscopy Investigations

**Fig. 4:**
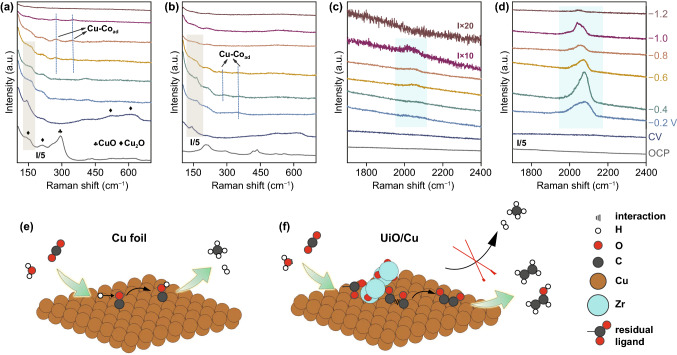
In situ Raman spectra recorded between 100 and 700 cm^−1^ on **a** UiO/Cu-bare and **b** UiO/Cu, and In situ Raman spectra recorded between 1700 and 2400 cm^−1^. on **c** UiO/Cu-bare and **d** UiO/Cu at OCP, after CV, and selected potential range from − 0.2 to − 1.2 V versus RHE with a potential interval of 0.2 V for 10 min. Schematic diagram of CO_2_RR process **e** on Cu and **f** on UiO/Cu

The modification of UiO-66 on Cu foil may regulate the CO_2_RR process by the following two possible mechanisms: (i) in situ electroreduction formed a-ZrO_x_ may stabilize Cu^+^ sites, and thus enhance the selectivity toward C2+ products; (ii) the loss of ligands of UiO-66 under negative potential may expose a large amount of coordination-unsaturated Zr sites, which can interact with CO_2_ molecule and steer adsorption mode of CO* intermediates, and thus promote the CO_2_ activation and C2+ products formation. To investigate the real reaction mechanism of CO_2_RR on UiO-66 modified Cu electrode, in situ surface-enhanced Raman spectroscopy was carried out. Figure 4a–b displayed the Raman bands between 100 and 700 cm^−1^, where the signals can be assigned to CuO_x_ and UiO-66 [65, 66]. For UiO/Cu-bare and UiO/Cu, a similar evolution trend of Cu_2_O was observed under CO_2_ electrolysis. When a negative potential was applied, the pristine CuO_x_ signals existing at the open-circuit potential (OCP) became weakened. However, the weak signals for CuO_x_ persisted under a wide potential window without obvious discrepancy on UiO/Cu-bare and UiO/Cu. In addition, three bands assigned to UiO-66 between 100 and 700 cm^−1^ at OCP on UiO/Cu disappeared under negative potential, in line with the decomposition of UiO-66 revealed by the ex situ characterizations. There is no indication that UiO-66 or a-ZrO_x_ could stabilize the Cu^+^ species under CO_2_RR condition. We thus exclude the possibility that the enhancement of C2+ selectivity comes from the stabilization of Cu^+^.

After ruling out the effect of Cu^+^, in situ surface-enhanced Raman spectroscopy also helped to explore the evolution of adsorption mode of CO_2_RR intermediate during CO_2_ electrolysis. The Raman bands at 283, 350 and 2070 cm^−1^ are assigned to the restricted rotation of adsorbed CO* on Cu, Cu−CO stretching, and atop C≡O stretching (CO_atop_), respectively. A visible difference of CO* related peaks can be observed on Cu foil, UiO/Cu-bare, and UiO/Cu. For Cu foil, the signals of CO* at low frequencies between 200 and 400 cm^−1^ cannot be observed (Fig. S13a). The CO_atop_ signal appeared at − 0.4 V versus RHE on Cu foil, which was less discernible compared with UiO/Cu-bare and UiO/Cu (Fig. S13b). As depicted in Fig. 4a–b, the restricted rotation of adsorbed CO* on Cu and Cu−CO stretching appeared at − 0.6 V versus RHE on UiO/Cu-bare, which is more negative than that on UiO/Cu (− 0.2 V vs. RHE). In Fig. 4c–d, the signal of CO_atop_ at 2070 cm^−1^ exhibited a similar convex trend on UiO/Cu-bare and UiO/Cu as the potential shifted negatively, probably corresponding to the accumulation and consumption of CO* [67]. Interestingly, the intensity of CO_atop_ signal on UiO/Cu was much higher than that on UiO/Cu-bare, indicating that UiO-66-derived a-ZrO_2_ can stabilize atop-bound CO* intermediates, which are generally believed to promote the C–C coupling process [68].

On the basis of our spectroscopic results and literature reports, we speculated that the relatively low CO* density on the surface hinders the CO* coupling process on Cu electrode (Fig. 4e). However, the in situ formed a-ZrO_x_ with abundant Lewis-acidic sites may activate inert CO_2_ and stabilize CO* intermediates by Lewis acid–base interaction [29, 38, 39], thus improving the probability of CO* coupling and producing more multi-carbon products (Fig. 4f). Nevertheless, more detailed experimental characterizations and/or computational efforts would be necessary to fully understand the mechanism.

## Conclusions

In summary, we report that Cu electrodes modified with UiO-66 nanoparticles would catalyze the CO_2_ electroreduction into C2+ products with enhanced activity and selectivity. The UiO-66 nanoparticles would decompose into amorphous ZrO_x_ with abundant Lewis-acidic sites during electrolysis, in situ constructing an amorphous ZrO_x_/Cu hetero-interface as dual-site catalyst. The optimal UiO-66-modified Cu electrode delivered an excellent selectivity of 74% for C2+ products, which was about 3.6 times higher than that for Cu electrode, as well as superior durability up to 32 h. In situ surface-enhanced Raman spectra indicate that the UiO-66-derived a-ZrO_x_ coating can promote the adsorption of atop-bound *CO intermediate, and thus facilitate the C–C coupling process. Constructing a robust hetero-interface opens up a new route to steer the adsorbed mode of intermediate and thus modulate the product selectivity. We believe that such a strategy would shed light on the design of other advanced electrocatalysts for efficient CO_2_RR.

## Supplementary Information

Below is the link to the electronic supplementary material.

Supplementary file1 (PDF 1134 kb)
