# Supplementary material for: Hetero-Interfaces on Cu Electrode for Enhanced Electrochemical Conversion of CO2 to Multi-Carbon Products
Source: Nanomicro Lett. 2022 Jun 14;14:134. doi: 10.1007/s40820-022-00879-5 (PMC9198171; doi:10.1007/s40820-022-00879-5)
Supplement: Supplementary file 1 — Supplementary file1 (PDF 1134 kb) [file 40820_2022_879_MOESM1_ESM.pdf]

Supporting Information for

Hetero-Interfaces on Cu Electrode for Enhanced Electrochemical Conversion of CO_2 to Multi-Carbon Products

Xiaotong Li[1,#], Jianghao Wang[1,#], Xiangzhou Lv[1], Yue Yang[1], Yifei Xu[1], Qian Liu[1], Hao Bin Wu[1,*]

[1] Institute for Composites Science Innovation (InCSI) and State Key Laboratory of Silicon Materials, School of Materials Science and Engineering, Zhejiang University, Hangzhou 310027, P. R. China

[#] Xiaotong Li and Jianghao Wang contributed equally to this work.

*Corresponding author. E-mail: hbwu@zju.edu.cn (Hao Bin Wu)

Supplementary Figures and Tables

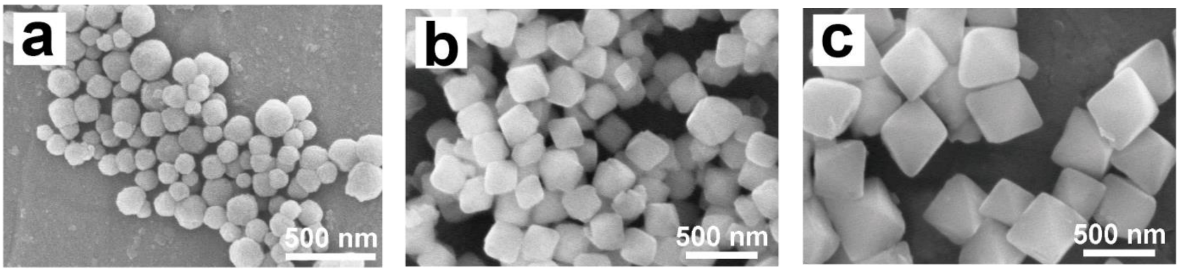

Fig. S1 SEM images of UiO-66 with different sizes of (**a**) 100 nm, (**b**) 300 nm, and (**c**) 600 nm

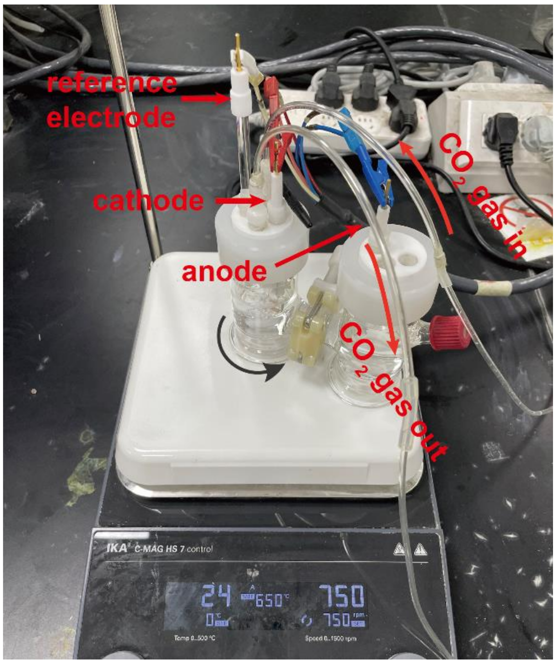

Fig. S2 Photo of the H-cell setup used in this work

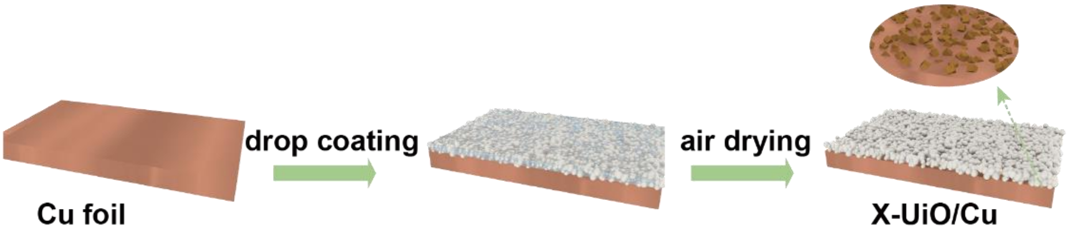

Fig. S3 Schematic of the synthetic process of UiO-66 modified Cu foil (X-UiO/Cu)

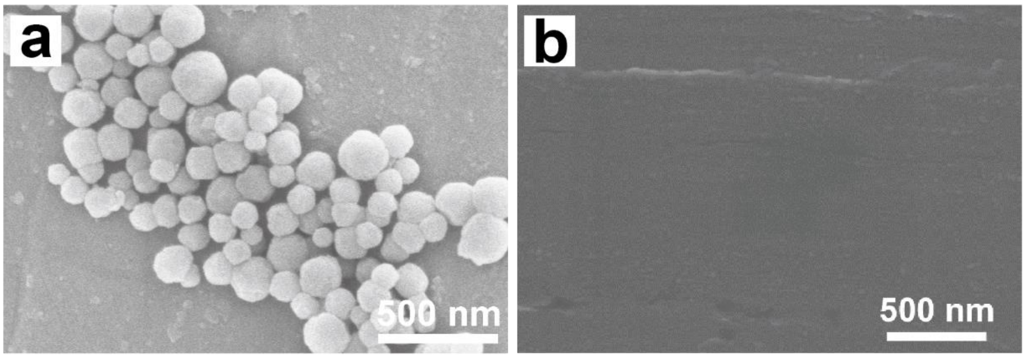

Fig. S4 SEM images of (**a**) UiO-66 nanoparticles and (**b**) mechanically polished Cu foil

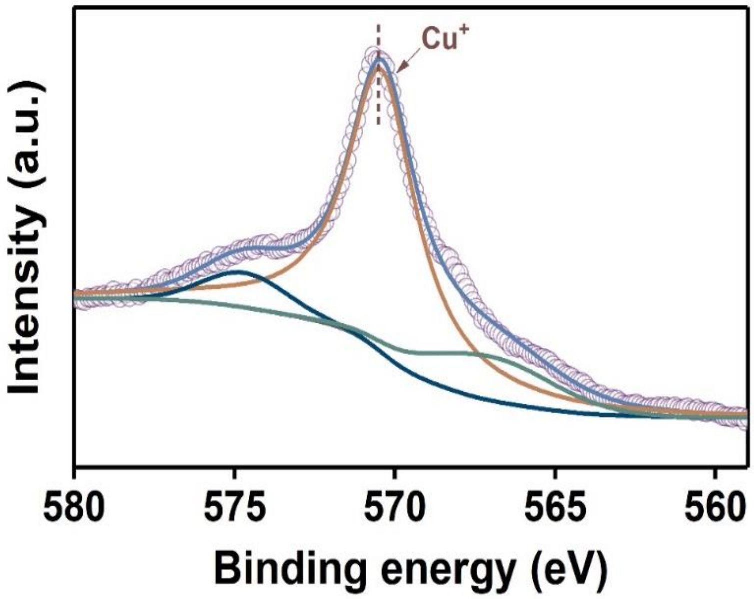

Fig. S5 Cu LMM AES spectrum of 0.5-UiO/Cu-bare before CO_2RR

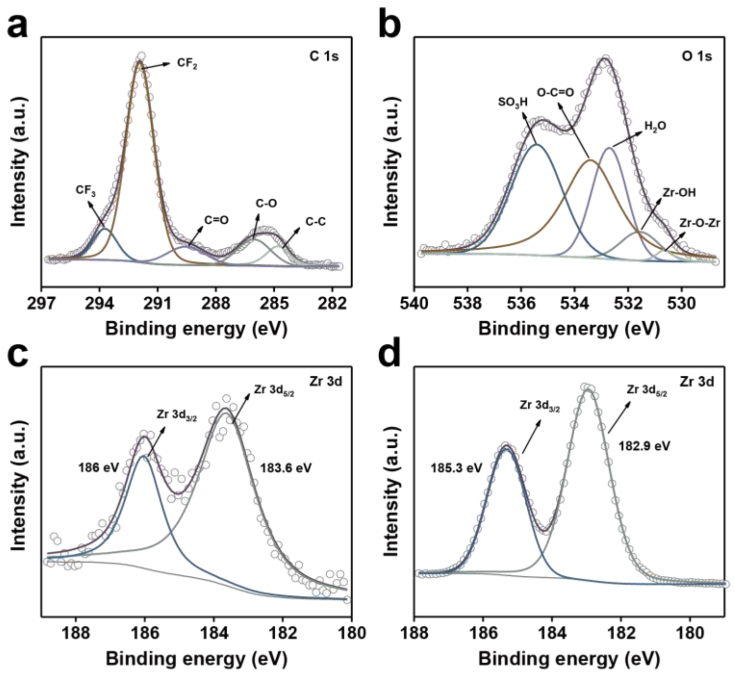

Fig. S6 (**a**) C 1s XPS spectrum, (**b**) O 1s XPS spectrum and (**c**) Zr 3d XPS spectrum of 0.5-UiO/Cu. (**d**) Zr 3d XPS spectrum of UiO-66 nanoparticles. All samples in Fig. S6 are characterized before CO_2RR

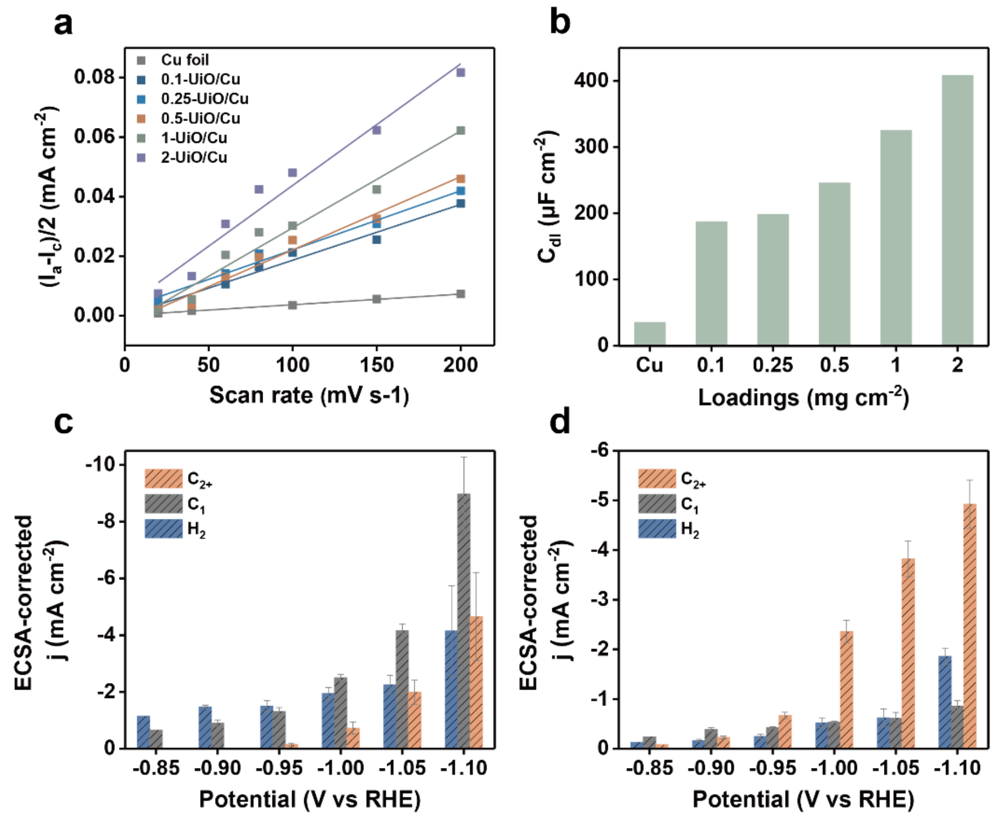

Fig. S7 (**a**) Linear fitting of capacitive currents and (**b**) the corresponding electrochemical double-layer capacitance (C_{dl}) (the slopes of the fitting curves in (**a**)) of X-UiO/Cu. The ECSA-corrected current density for H_2, C_1, and C_{2+} products as a function of potential on (**c**) Cu and (**d**) 0.5-UiO/Cu

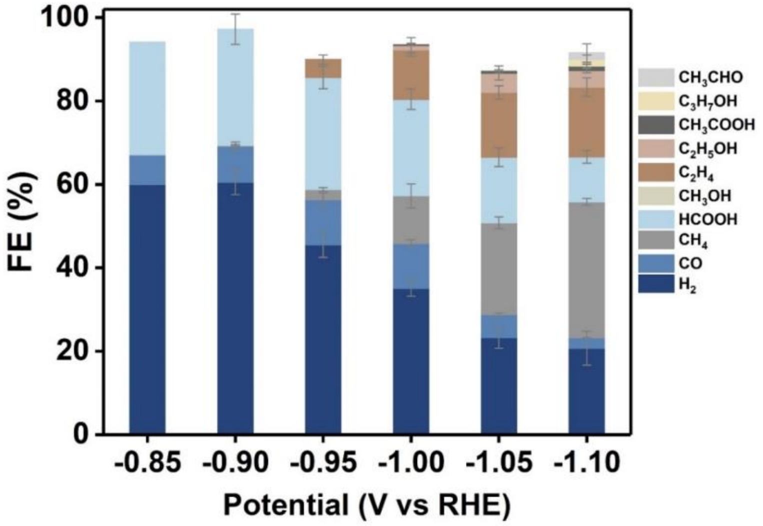

Fig. S8 FEs of CO_2RR and HER products on Cu foil as a function of potential

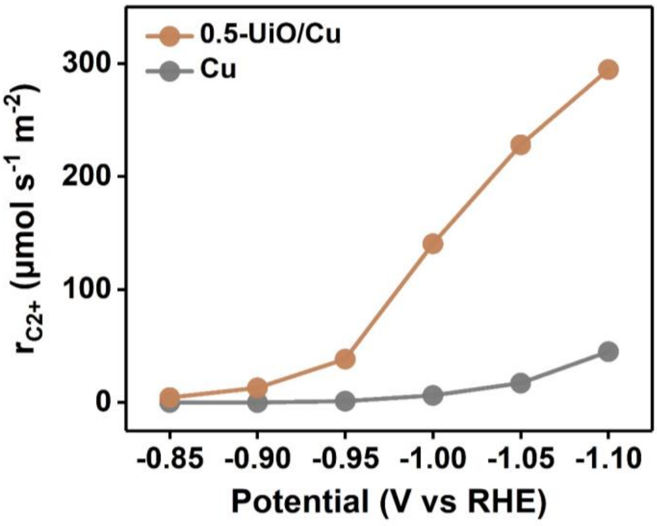

Fig. S9 Formation rates of C2+ products (r_{C2+}) on Cu foil and 0.5-UiO/Cu as a function of potential

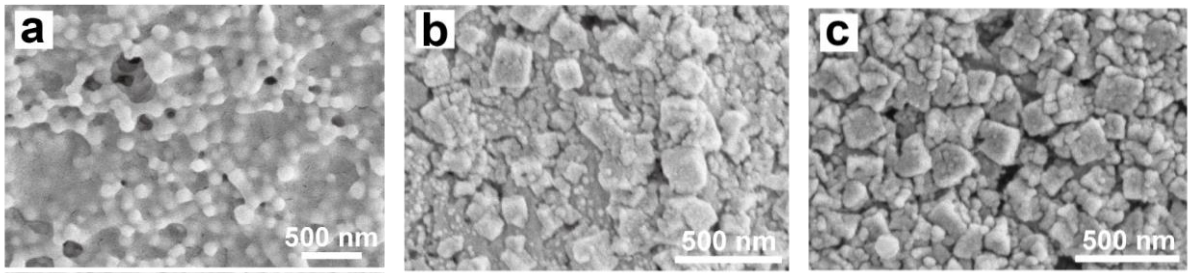

Fig. S10 Top-view SEM images of 0.5-UiO/Cu electrode after CV: (**a**) with and (**b**) without the surface coating layer. (**c**) Top-view SEM image of 0.5-UiO/Cu electrode after CA without the surface coating layer

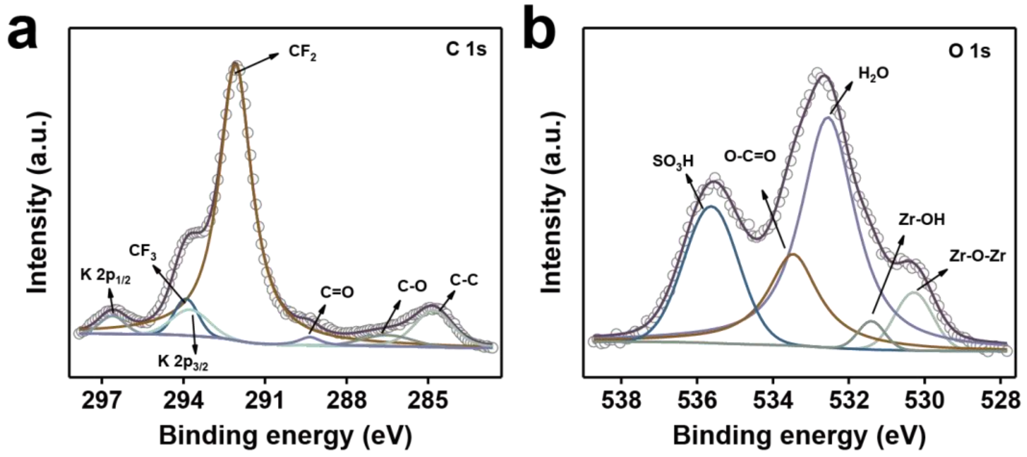

Fig. S11 (**a**) C 1s spectrum and (**b**) O 1s spectrum of 0.5-UiO/Cu after CO_2RR at -1.05 V vs. RHE for 1 h

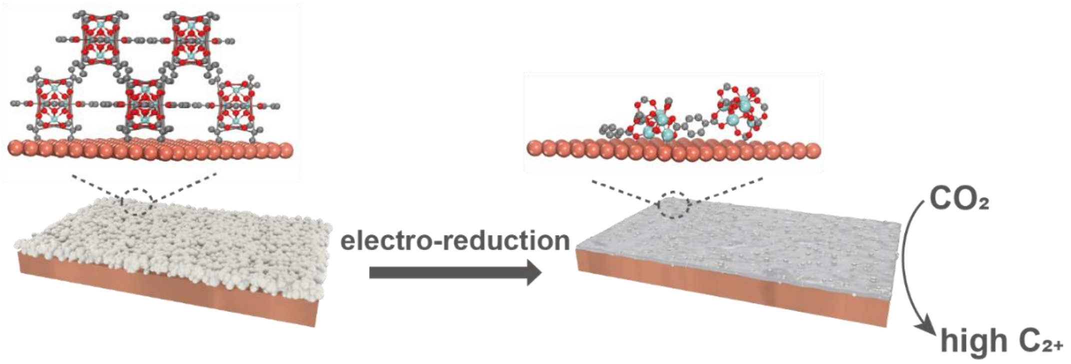

Fig. S12 Schematic of possible evolution process of UiO-66 coating on X-UiO/Cu electrode under CO_2RR

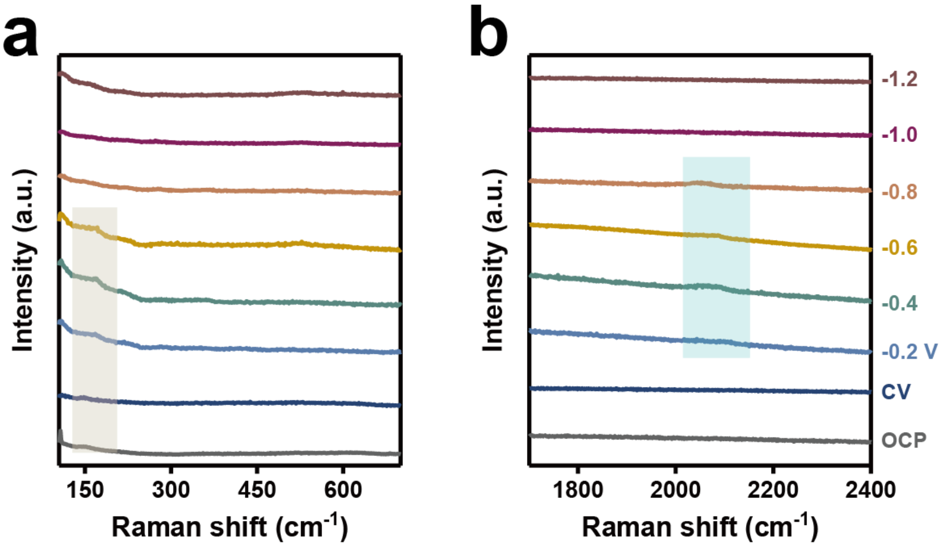

Fig. S13 The *in situ* surface-enhanced Raman spectra recorded between (**a**) 100-700 cm^{-1} and (**b**) 1700-2400 cm^{-1} on Cu foil at OPC, after CV, and at the selected potential range of -0.2 V to -1.2 V vs. RHE with a potential interval of 0.2 V for 10 min

Table S1 The C_{dl}, R_f, *ECSA*, and *ECSA*-corrected j_{C2+} of Cu and X-UiO/Cu

Sample	C_{dl} (μF cm^{-2})	R_f	*ECSA*	*ECSA*-corrected j_{C2+}
Cu	35.37	1	1	1.99
0.1-UiO/Cu	187.39	5.3	5.3	1.76
0.25-UiO/Cu	198.41	5.61	5.61	3.35
0.5-UiO/Cu	245.9	6.95	6.95	3.82
1-UiO/Cu	325.32	9.2	9.2	2.90
2-UiO/Cu	408.26	11.54	11.54	2.45

Table S2 Comparison of CO_2RR performance on 0.5-UiO/Cu with state-of-the-art Cu-based catalyst evaluated in H-type cell

Refs.	catalyst	cell type	electrolyte	E_{we}/(RHE)	FE of C_{2+}	ECSA-collected jC_{2+} (mA cm^{-2})	geometric area normalized jC_{2+} (mA cm^{-2})	r_{C2+}: Formation rate of C2+ (μmol s^{-1} m^{-2})	stability (h)
**This work**	**0.5-UiO/Cu**	**H-cell**	**0.1 M $KHCO_3$**	**-1.05**	**74.17%**	**-3.822**	**-26.57**	228.08	**32**
ACS Catal. 2021, 11, 2473-2482	Cu@N_xC	home-made H-cell	0.1 M $KHCO_3$	-1.1	76.8%	-5	-14.9	128.69	2.7
Joule. 2021 5, 429-440	Cu-DS	H-cell	0.1 M $KHCO_3$	-1.08	78%	N/A	-23.4	186.56	30
Small. 2021, 2102293	Cu@Ag-2	Flow-cell	1 M KOH	-1.1	67.6%	N/A	-22.7	193.53	14
Nano Res. 2021 doi.org/10.1007/s12274-021-3532-7	Cu-s	H-cell	0.1 M $KHCO_3$	-1.1	55.8%	N/A	-26.69	230.52	8
Electrochim Acta. 2021, 388, 138552	p-Cu	H-cell	0.1 M $KHCO_3$	-1.3	57.2%	N/A	-22.65	195.69	10
Green Chem. 2020, 22, 6540-6546	CuO-CeO_2/CB	H-cell	0.1 M $KHCO_3$	-1.1	50%	N/A	-3.77	32.56	9
Angew. Chem. Int. Ed. 2021, 60, 7426-7435	5-Ag/Cu_2O	H-cell	0.1 M $KHCO_3$	-0.98	65%	-0.41	-6.09	51.25	12
Angew. Chem. Int. Ed. 2021, 60, 15344-15347	Cu/$CuSiO_3$	H-cell	0.1 M $KHCO_3$	-1.1	60.64%	N/A	-12.25	105.80	6
ACS Appl. Nano Mater. 2020, 3, 257-263	Cu GNC-VL	H-cell	0.5 M $KHCO_3$	-0.87	70.5%	N/A	-7.33	63.33	12
Chem. Mater. 2020, 32, 3304-3311	Cu_3N	H-cell	0.1 M $CsHCO_3$	-1	68%	-0.714	-12.58	102.15	3.33
ACS Energy Lett. 2021, 6, 437-444	CuBr-DDT	H-cell	0.1 M KCl	-1.25	72%	-8.75	-9.02	76.90	15
ACS Catal. 2020, 10, 4103-4111	Cu/PANI	H-cell	0.1 M $KHCO_3$	-1.2	66%	-5.17	-14.9	127.91	20

Some data in **Table S2** is collected from figures in the corresponding literature, which may be less precise.

Table S3 EDS results of 0.5-UiO/Cu NPs, 0.5-UiO/Cu-CV NPs, and 0.5-UiO/Cu-CA NPs

trail/at%	C	O	Cu	Zr
UiO/Cu NPs	67.89	27.47	0.83	3.80
UiO/Cu-CV NPs	49.71	41.86	1.04	7.39
UiO/Cu-CA NPs	33.71	49.26	2.72	14.32

Table S4 FEs of H_2 and various CO_2RR products as well as the geometric current density on Cu foil as a function of potential

E_{we} (RHE)	j (mA cm^{-2})	H_2	CO	CH_4	HCOOH	C_2H_4	C_2H_5OH	CH_3COOH	C_3H_7OH	CH_3CHO
-0.85	1.92	60.01	7.12	0	27	0	0	0	0	0
-0.90	2.45	60.5±2.91	8.69±1.03	0.18±0.18	27.84±3.66	0	0	0	0	0
-0.95	3.29	45.49±2.97	10.86±1.62	2.46±0.44	26.85±2.72	4.3±1.13	0	0	0	0
-1	5.53	35.14±1.99	10.72±0.89	11.4±2.85	23.17±2.44	11.88±1.53	1.06±1.83	0.14±0.25	0	0
-1.05	9.63	23.3±2.52	5.52±0.38	22.01±1.44	15.73±2.22	15.5±1.57	4.66±1.72	0.41±0.36	0	0
-1.1	19.67	20.72±4.04	2.52±0.06	32.59±0.83	10.79±1.48	16.72±2.19	3.99±0.49	1.09±0.40	1.56±1.08	1.57±2.22

Table S5 FEs of H_2 and various CO_2RR products as well as the geometric current density on 0.5-UiO/Cu electrode as a function of potential

E_{we} (RHE)	j (mA cm^{-2})	H_2	CO	CH_4	HCOOH	CH_3OH	C_2H_4	C_2H_5OH	CH_3COOH	C_3H_7OH	CH_3CHO
-0.85	3.06	29.16	19.1	0	36.58	0	7.18	7	0	3.94	0
-0.9	5.81	19.94±2.39	17.68±0.91	0	28.73±2.69	0	15.29±1.12	7.07±3.12	0	5.25±0.62	0
-0.95	9.72	17.76±3.47	10.5±1.2	1.33±0.31	18.01±1.91	0.84±0.78	28.44±2.47	9.63±1.02	0.54±0.19	8.68±0.79	0.56±0.47
-1	23.95	15.06±1.85	3.15±0.28	3.38±0.58	8.73±0.99	0.58±0.25	40.68±1.4	16.8±1.33	0.75±0.42	8.21±0.31	1.94±0.93
-1.05	35.78	11.98±2.81	1.41±0.28	4.45±1	5.7±2.07	0.53±0.58	42.2±1.92	20.97±0.73	0.86±0.21	8.2±0.25	1.95±0.63
-1.1	56.71	22.88±1.48	0.83±0.19	6.35±0.9	3.06±0.55	0.5±0.36	33.02±1.77	22.41±2.44	0.5±0.19	3.77±0.74	0.75±0.05

Table S6 FEs of H_2 and various CO_2RR products as well as the geometric current density on various X-UiO/Cu electrodes at -1.05 V vs. RHE

Loading (mg cm^{-2})	j (mA cm^{-2})	H_2	CO	CH_4	HCOOH	CH_3OH	C_2H_4	C_2H_5OH	CH_3COOH	C_3H_7OH	CH_3CHO
0.1	16.5	15.84	2.25	9.36	10.66	0	33.3	16.32	0.13	5.88	0.92
0.25	26.34	9.45±0.9	1.66±0.14	5.56±0.62	5.91±0.94	0.17±0.3	43.32±2.01	18.39±0.94	0.72±0.18	6.58±0.28	1.77±0.51
0.5	35.78	11.98±2.81	1.41±0.28	4.45±1	5.7±2.07	0.53±0.58	42.2±1.92	20.97±0.73	0.86±0.21	8.2±0.25	1.95±0.63
1	44.34	22.56±2.93	1.49±0.16	4.8±0.97	5.84±1.23	0.7±0.82	34.11±1.6	17.61±1.7	0.75±0.24	6.27±2.94	1.22±0.59
2	56.72	27.36	1.08	4.58	6.08	0.78	27.67	13.3	0.43	8.2	0.32
